# Maternal Humoral Immune Responses Do Not Predict Postnatal HIV-1 Transmission Risk in Antiretroviral-Treated Mothers from the IMPAACT PROMISE Study

**DOI:** 10.1128/mSphere.00716-19

**Published:** 2019-10-23

**Authors:** Eliza D. Hompe, Denise L. Jacobson, Joshua A. Eudailey, Kevin Butler, Whitney Edwards, Justin Pollara, Sean S. Brummel, Genevieve G. Fouda, Lameck Chinula, Melvin Kamanga, Aarti Kinikar, Dhayendre Moodley, Maxensia Owor, Mary Glenn Fowler, Sallie R. Permar

**Affiliations:** a Duke University School of Medicine, Durham, North Carolina, USA; b Center for Biostatistics in AIDS Research, Harvard T. H. Chan School of Public Health, Boston, Massachusetts, USA; c Duke Human Vaccine Institute, Duke University School of Medicine, Durham, North Carolina, USA; d Department of Surgery, Duke University School of Medicine, Durham, North Carolina, USA; e Department of Pediatrics, Duke University School of Medicine, Durham, North Carolina, USA; f University of North Carolina Project-Malawi, Lilongwe, Malawi; g Johns Hopkins University Research Project, Blantyre, Malawi; h Byramjee Jeejeebhoy Government Medical College, Pune, Maharashtra, India; i Centre for the AIDS Programme of Research in South Africa and School of Clinical Medicine, College of Health Sciences, University of KwaZulu Natal, Durban, South Africa; j Johns Hopkins University Research Collaboration, Makerere University, Kampala, Uganda; k Department of Pathology, Johns Hopkins University School of Medicine, Baltimore, Maryland, USA; University of Maryland School of Medicine

**Keywords:** ADCC, HIV-1, antibodies, antiretroviral therapy, breast milk, postnatal transmission

## Abstract

To design immune interventions that can synergize with antiretroviral therapy (ART) to reduce the rate of HIV mother-to-child transmission (MTCT), it is essential to characterize maternal immune responses in the setting of ART during pregnancy and breastfeeding and define their effect on MTCT. Prior studies reported an association between breast milk envelope (Env)-specific antibodies and antibody-dependent cell cytotoxicity (ADCC) activity with reduced postnatal transmission. In this study, we investigated whether these immune correlates were similarly associated with protection in a matched case-control study of mother-infant pairs receiving maternal ART or infant nevirapine prophylaxis during breastfeeding in the International Maternal-Pediatric-Adolescent AIDS Clinical Trials Network Promoting Maternal-Infant Survival Everywhere (PROMISE) trial, assessing postnatal transmission risk in 19 transmitting and 57 nontransmitting mothers using conditional logistic regression models adjusted for maternal plasma viral load. The odds ratios of postnatal MTCT for a 1-unit increase in an immune correlate were 3.61 (95% confidence interval [CI], 0.56, 23.14) for breast milk Env-specific secretory IgA (sIgA), 2.32 (95% CI, 0.43, 12.56) for breast milk and 2.16 (95% CI, 0.51, 9.14) for plasma Env-specific IgA, and 4.57 (95% CI, 0.68, 30.48) for breast milk and 0.96 (95% CI, 0.25, 3.67) for plasma ADCC activity, with all CIs spanning 1.0. Interestingly, although mucosal IgA responses are poor in untreated HIV-infected women, there was a strong correlation between the magnitudes of breast milk and plasma Env-specific IgA in this cohort. In this analysis of the small number of postnatal virus transmissions in the landmark PROMISE study, no single antibody response was associated with breast milk transmission risk.

**IMPORTANCE** Each year, >150,000 infants become newly infected with HIV-1 through MTCT despite ART, with up to 42% of infections occurring during breastfeeding. Several factors contribute to continued pediatric infections, including ART nonadherence, the emergence of drug-resistant HIV strains, acute infection during breastfeeding, and poor access to ART in resource-limited areas. A better understanding of the maternal humoral immune responses that provide protection against postnatal transmission in the setting of ART is critical to guide the design of maternal vaccine strategies to further eliminate postnatal HIV transmission. In this study, we found that in women treated with antiretrovirals during pregnancy, there was a positive correlation between plasma viral load and breast milk and plasma IgA responses; however, conclusions regarding odds of MTCT risk were limited by the small sample size. These findings will inform future studies to investigate maternal immune interventions that can synergize with ART to eliminate MTCT during breastfeeding.

## Abstract (precis)

Each year, >150,000 infants become newly infected with HIV-1 through MTCT despite ART, with up to 42% of infections occurring during breastfeeding. Several factors contribute to continued pediatric infections, including ART nonadherence, the emergence of drug-resistant HIV strains, acute infection during breastfeeding, and poor access to ART in resource-limited areas. A better understanding of the maternal humoral immune responses that provide protection against postnatal transmission in the setting of ART is critical to guide the design of maternal vaccine strategies to further eliminate postnatal HIV transmission. In this study, we found that in women treated with antiretrovirals during pregnancy, there was a positive correlation between plasma viral load and breast milk and plasma IgA responses; however, conclusions regarding odds of MTCT risk were limited by the small sample size. These findings will inform future studies to investigate maternal immune interventions that can synergize with ART to eliminate MTCT during breastfeeding.

## INTRODUCTION

With optimal maternal antiretroviral therapy (ART) and infant prophylaxis, the rate of human immunodeficiency virus type 1 (HIV-1) transmission through breastfeeding has been significantly reduced, to less than 5% (1–5). However, several factors contribute to continued infant exposure to HIV during breastfeeding and thus a risk of vertical transmission, including nonadherence to ART, acute maternal infections, and loss to care, primarily in resource-limited areas (6). Postnatal HIV-1 transmission accounts for up to 42% of the overall mother-to-child transmission (MTCT) rate, and the risk of transmission remains constant for the duration of breastfeeding (7, 8).

In addition to high maternal plasma viral load and low peripheral CD4^+^ T cell count, risk factors specific to breast milk transmission include breast milk viral load and breast pathologies, including mastitis or abscess (8–10). As a result, strategies to prevent transmission focusing on avoidance or early cessation of breastfeeding have been tested. Yet formula feeding is associated with increased infant morbidity due to diarrheal illnesses and poor nutrition in developing countries (11, 12). Moreover, early weaning to decrease the duration of breastfeeding, and thus infant exposure to HIV-1, led to higher mortality in HIV-infected infants (13). Infants receiving mixed feeding compared to exclusively breastfed infants are also at increased risk of postnatal HIV-1 transmission and have higher mortality in the first few months of life (14, 15). As a result, current World Health Organization guidelines recommend universal breastfeeding in HIV-infected mothers in resource-limited regions (16). Therefore, alternative interventions to avoid transmission of HIV through breastfeeding will be needed to eliminate postnatal MTCT.

Even in the absence of maternal ART, the majority of HIV-exposed, breastfeeding infants remain uninfected, suggesting that there may be maternal humoral immune responses in breast milk that protect against infant HIV-1 acquisition (17, 18). There are conflicting data on the role of virus envelope (Env)-specific antibodies (Abs) in breast milk in protection against postpartum transmission. Several studies have found no association between HIV-1 Env-specific Abs and risk of postpartum transmission (19, 20). One group reported that Env-specific secretory IgA (sIgA) was detected more frequently in transmitting than in nontransmitting mothers, suggesting that it is not protective and may instead be associated with increased transmission risk (21). Further work has evaluated the role of neutralizing and antibody-dependent cell-mediated cytotoxicity (ADCC) activity in breast milk in transmitting and nontransmitting mothers (22). While there are low levels of neutralizing antibodies in breast milk of all HIV-infected mothers, high ADCC activity has been reported in nontransmitting compared to transmitting mothers (22). These findings suggest that nonneutralizing antibody functions in breast milk may help reduce postnatal transmission risk and merit further investigation in larger immune correlate studies of postnatal MTCT.

Our group recently investigated the immune correlates of postnatal transmission of HIV-1 in a cohort of clade C HIV-1-infected transmitting (*n* = 22) and matched nontransmitting (*n* = 65) women from the control arm of the Malawian Breastfeeding, Antiretrovirals, and Nutrition (BAN) clinical trial (23). Of note, these mothers only received postnatal ART prophylaxis around delivery, providing an opportunity to probe the relationship between natural immunologic factors and postnatal transmission risk. Importantly, the study identified that the magnitude of Env-specific IgA and sIgA responses in breast milk directed against a consensus Env gp140 antigen were significantly associated with reduced risk of postnatal HIV-1 transmission (23). These findings indicate that a viable strategy for decreasing postnatal transmission of HIV-1 to infants could be a maternal vaccine that boosts Env-specific IgA responses in breast milk. However, as the mothers in the BAN cohort did not receive postnatal ART, the current standard of care, it is important to investigate whether these previously defined breast milk immune correlates are important for protection in mother-infant pairs receiving postnatal prophylaxis.

In this study, we aimed to determine whether breast milk Env-specific IgA and sIgA are similarly predictive of transmission risk in the setting of maternal ART or infant nevirapine (NVP) prophylaxis during breastfeeding. We studied a cohort of postnatally transmitting (*n* = 19) and nontransmitting (*n* = 57) mothers from the breastfeeding arm of the International Maternal-Pediatric-Adolescent AIDS Clinical Trials (IMPAACT) network Promoting Maternal-Infant Survival Everywhere (PROMISE) 1077BF (Breastfeeding) protocol (24). Postnatal transmission rates in the postpartum component of the PROMISE trial were 0.6% and 0.9% for the maternal ART and infant NVP arms, respectively (5). We assessed levels of maternal HIV Env-binding and functional plasma and breast milk antibody responses and their association with postnatal HIV-1 transmission. This work provides valuable insight into the role of maternal Env-specific HIV-1 antibody responses in postnatal transmission with standard-of-care therapy. Furthermore, it presents a novel characterization of the magnitude and breadth of immune responses across breast milk and plasma in women who received ART during pregnancy, which has not previously been described. This study will inform the design of maternal immune interventions that can synergize with ART to prevent MTCT and improve the safety of breastfeeding.

## RESULTS

### High plasma viral loads and lower peripheral CD4^+^ T cell counts in postnatally transmitting compared to those in nontransmitting mothers.

Transmitting and nontransmitting mothers were similar on age, gestational age of the infant at birth, sex of the infant, and postpartum randomization to maternal ART or infant NVP prophylaxis (Table 1). However, of note, transmitting women had higher plasma viral loads at the time of measurement closest to transmission than did matched nontransmitting women {median [quartile 1 (Q1) to quartile 3 (Q3) of the interquartile range], 4.20 [3.24 to 4.96] versus 2.15 [1.59 to 3.31] log_10_ copies/ml}. In addition, 89% of transmitting women had a plasma viral load of >1,000 copies/ml prior to transmission, compared to only 35% of the nontransmitting women. Transmitting women also had lower CD4^+^ T cell counts than nontransmitting women (median [Q1 to Q3], 563 [425 to 839] versus 743 [621 to 935] cells/mm^3^). Finally, transmitting women had fewer previous births than did nontransmitting women (median maternal parity of 2 versus 5, including PROMISE delivery).

**TABLE 1:** Clinical characteristics of postnatal transmitting and matched nontransmitting HIV-1-infected mothers from the PROMISE 1077BF study

Characteristic^*a*^	Values for subjects with indicated transmission status	
	Transmitter (*n* = 19)	Nontransmitter (*n* = 57)
Maternal age (yrs)		
Min–max	18–38	18–36
Median (Q1–Q3)	24 (23–29)	26 (23–29)
Maternal viral load (log_10_ copies/ml), first measurement after delivery		
Min–max	1.48–5.57	1.30–5.25
Median (Q1–Q3)	3.97 (2.52–5.00)	2.22 (1.60–2.68)
Undetectable	3 (16%)	17 (30%)
Detectable and <1,000 copies/ml	3 (16%)	29 (51%)
≥1,000 copies/ml	13 (68%)	11 (19%)
Maternal viral load (log_10_ copies/ml), measurement closest to transmission		
Min–max	1.59–5.47	1.28–5.25
Median (Q1–Q3)	4.20 (3.24–4.96)	2.15 (1.59–3.31)
Undetectable	1 (5%)	24 (42%)
Detectable and <1,000 copies/ml	1 (5%)	13 (23%)
≥1,000 copies/ml	17 (89%)	20 (35%)
Maternal peripheral CD4^+^ T cell count (cells/mm^3^), measurement after delivery		
Min–max	188–1,337	366–1,606
Median (Q1–Q3)	563 (425–839)	743 (621–935)
Maternal peripheral CD4^+^ T cell count (cells/mm^3^), closest to transmission		
Min–max	270–1,337	314–1,606
Median (Q1–Q3)	560 (436–795)	743 (628–966)
Gestational age at birth (wks)		
Min–max	35–41	30–48
Median (Q1–Q3)	38 (36–40)	38 (35–39)
Sex of child		
M	8 (42%)	22 (39%)
F	11 (58%)	35 (61%)
Maternal parity, including PROMISE delivery		
Min–max	1–5	1–9
Median (Q1–Q3)	2 (2–3)	5 (4–6)
Country		
India	1 (5%)	3 (5%)
Malawi	12 (63%)	36 (63%)
South Africa	2 (11%)	6 (11%)
Uganda	4 (21%)	12 (21%)
PP component randomization^*b*^		
Maternal triple ART	8 (42%)	26 (46%)
Infant prophylaxis	6 (32%)	26 (46%)
AP observation follow-up	5 (26%)	5 (9%)
Age of infant at breast milk specimen collection prior to transmission (wks)		
Min–max	1–74	1–74
Median (Q1–Q3)	26 (6–50)	26 (6–50)
Infant age at infection (wks)		
Min–max	6–87	
Median (Q1–Q3)	38 (14–74)	

a Min, minimum; max, maximum; M, male; F, female.

b In the maternal triple-ART arm, the mother received lopinavir-ritonavir (LPV-RTV) plus tenofovir-emtricitabine (TDF/FTC). In the infant prophylaxis arm, the infant received nevirapine (NVP). In the AP observation follow-up, the mother received a triple-ART regimen until the week 1 postpartum visit.

### Maternal Env-specific IgA levels and ADCC activity are correlated across breast milk and plasma compartments.

While Env-specific IgG and ADCC activity in plasma and breast milk are consistently directly correlated, we have previously described a lack of correlation between HIV-1 Env-binding IgA responses in breast milk and plasma (25). We examined correlations between IgA and ADCC responses across breast milk and plasma compartments (Table 2). The magnitude of total IgA binding responses against clade B HIV-1 consensus gp140 antigen (B.con gp140) was positively correlated between breast milk and plasma (ρ = 0.683 and *P* < 0.001). ADCC antibody titer and potency were weakly correlated across the two compartments (ρ = 0.317 and *P* = 0.005 for ADCC antibody titer; ρ = 0.315 and *P* = 0.006 for potency).

**TABLE 2:** Correlations of primary and secondary maternal immune variables across breast milk and plasma compartments

Immune response: breast milk vs plasma	Spearman correlation coefficient (*P* value)
Total IgA against HIV-1 B.con gp140	0.683 (<0.001)
ADCC antibody titer	0.317 (0.005)
ADCC potency (maximum % killing)	0.315 (0.006)

### Primary analysis: breast milk Env-specific IgA and sIgA binding response and ADCC antibody titer association with postnatal transmission risk.

**FIG 1:**
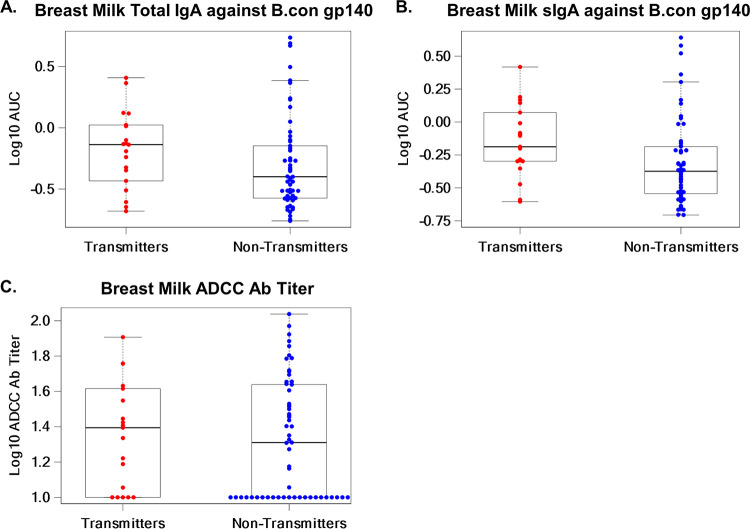
Magnitudes of breast milk Env-specific IgA and sIgA binding responses by ELISA and functional ADCC antibody titers in transmitting and nontransmitting mothers. The magnitude of breast milk total IgA (A) and sIgA (B) binding to B.con gp140, measured as the area under the curve by ELISA, was higher in transmitting than in nontransmitting women (median log_10_ AUC, −0.14 versus −0.40 and −0.19 versus −0.37). Breast milk ADCC Ab titer (C) was also higher in transmitting compared to nontransmitting women (median log_10_ ADCC Ab titer, 1.39 versus 1.31). Medians are represented by horizontal black lines. The box represents the interquartile range, and the dashed lines extend to the highest and lowest values.

We investigated whether breast milk total IgA and sIgA directed against B.con gp140 and breast milk ADCC responses were associated with postnatal transmission in this cohort. The odds ratio (OR) (95% confidence interval [CI], *P* value) of postnatal transmission for each 1-unit increase in breast milk total IgA against B.con gp140 was 2.32 (0.43, 12.56) and that for sIgA against B.con gp140 was 3.61 (0.56, 23.14) (Table 3). Env-specific binding Ab responses in breast milk had, on inspection, higher median levels in transmitting than in nontransmitting women (median log_10_ AUC [area under the curve], −0.14 versus −0.40 and −0.19 versus −0.37 for total IgA and sIgA, respectively) (Fig. 1A and B). A sizable proportion of mothers had undetectable functional ADCC activity in breast milk (5/19 [26%] of transmitting women and 23/57 [40%] of nontransmitting women), and the odds ratio of HIV-1 transmission for breast milk ADCC antibody titer was 4.57 (0.68, 30.48; *P* = 0.12) (Table 3 and Fig. 1C).

**TABLE 3:** Associations of HIV Env-specific breast milk and plasma antibody responses and HIV-1 transmission to the infant

Analysis	Immune response	Assay	Odds ratio (95% CI)^*a*^	*P* value
Primary immune variable	Breast milk total IgA against HIV-1 B.con gp140	ELISA	2.32 (0.43, 12.56)	0.33
	Breast milk sIgA against HIV-1 B.con gp140^*b*^	ELISA	3.61 (0.56, 23.14)	0.18
	Breast milk ADCC antibody titer^*b*^	ADCC-Luc	4.57 (0.68, 30.48)	0.12
				
Secondary immune variable	Plasma total IgA against HIV-1 B.con gp140	ELISA	2.16 (0.51, 9.14)	0.30
	Plasma ADCC antibody titer^*b*^	ADCC-Luc	0.96 (0.25, 3.67)	0.95
	Breast milk ADCC potency	ADCC-Luc	1.02 (0.95, 1.08)	0.61
	Plasma ADCC potency	ADCC-Luc	1.04 (0.96, 1.13)	0.30

a Odds ratios (ORs) and 95% confidence intervals (CI) for ELISA area under the curve and ADCC antibody titer and potency were determined by logistic regression modeling controlling for log_10_ plasma viral load. Odds ratios greater than 1 indicate higher odds of transmission for a 1-unit change in the immune response levels.

b Immune response is dichotomous, with categories of below the lower limit of detection (LLD) and above the LLD. The odds ratio interprets as above the LLD relative to below the LLD.

### Secondary analysis: plasma Env-specific IgA binding responses, plasma ADCC antibody titer, and plasma and breast milk ADCC potency association with MTCT risk.

**FIG 2:**
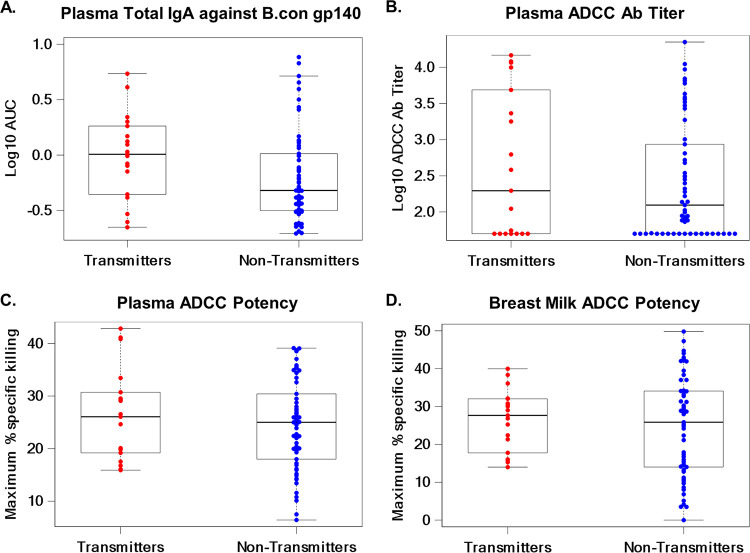
Magnitudes of plasma Env-specific IgA binding responses by ELISA, plasma ADCC antibody titer, and plasma and breast milk ADCC potency in transmitting and nontransmitting women. The magnitude of plasma total IgA (A) binding to B.con gp140, measured as the area under the curve by ELISA, was higher in transmitting than in nontransmitting women (median log_10_ AUC, 0.01 versus 0.48). Plasma ADCC Ab titer (B) was also higher in transmitting women than in nontransmitting women (median log_10_ ADCC Ab titer, 2.29 versus 2.09). Plasma (C) and breast milk (D) ADCC potencies, as maximum percent specific killing, were higher in transmitting than in nontransmitting women (median max percent specific killing, 26.06 versus 25.00, and 27.66 versus 25.86). Medians are represented by horizontal black lines. The box represents the interquartile range, and the dashed lines extend to the highest and lowest values.

A secondary analysis was conducted to determine if the magnitude of HIV Env-specific binding antibody responses in plasma, ADCC antibody titer in plasma, and ADCC potency in breast milk or plasma were associated with risk of postnatal HIV transmission. The odds ratios for postnatal transmission of HIV were 2.16 (0.51, 9.14) for plasma total IgA against B.con gp140, 0.96 (0.25, 3.67) for plasma ADCC antibody titer, and 1.04 (0.96, 1.13) for plasma ADCC potency (Table 3). In addition, for breast milk ADCC potency the odds ratio of HIV-1 transmission to the infant was 1.02 (0.95, 1.08) (Table 3). Interestingly, all of these immune responses had higher median levels in transmitting than in nontransmitting women (Fig. 2).

### Breast milk sIgA and plasma IgA responses correlate with maternal plasma viral load.

As transmitting women had higher median plasma viral loads than nontransmitting women (Table 1), we performed correlations between each antibody response and plasma viral load to assess whether this association could explain the higher median levels of immune correlates that we observed in transmitting than in nontransmitting women (Table 4). Maternal plasma viral load was positively correlated with breast milk sIgA (ρ = 0.248 and *P* = 0.03) and plasma total IgA binding to B.con gp140 (ρ = 0.294 and *P* = 0.01). Correlations were very weak between maternal viral load and all other measured immune parameters, including breast milk total IgA binding to B.con gp140, breast milk ADCC antibody titer and potency, and plasma ADCC antibody titer and potency (Table 4).

**TABLE 4:** Correlations of maternal plasma viral load and HIV Env-specific breast milk and plasma antibody responses and ADCC activity

Immune response	Spearman correlation coefficient (*P* value)
Breast milk total IgA against HIV-1 B.con env03 gp140 by ELISA	0.223 (0.053)
Breast milk sIgA against HIV-1 B.con env03 gp140 by ELISA	0.248 (0.030)
Breast milk ADCC antibody titer	−0.058 (0.62)
Plasma total IgA against HIV-1 B.con env03 gp140 by ELISA	0.294 (0.010)
Plasma ADCC antibody titer	0.073 (0.53)
Breast milk ADCC potency	0.026 (0.82)
Plasma ADCC potency	0.039 (0.74)

### Secondary analysis: breast milk and plasma Env epitope-specific binding antibody responses association with postnatal transmission risk.

In further secondary analysis, we assessed the magnitude of breast milk and plasma Env-specific binding antibody responses against a panel of HIV-1 Env antigens and their association with postnatal transmission risk (see Table S1 in the supplemental material). We measured Env-specific IgG and IgA responses in plasma and Env-specific IgG, IgA, and sIgA responses in breast milk by binding antibody multiplex assay (BAMA). Overall, the magnitude of these Env epitope-specific antibody responses in either compartment and transmission risk in the logistic regression model did not show any conclusive associations after correction for multiple comparisons (Table S1). Of note, the 95% confidence intervals in this analysis were large.

10.1128/mSphere.00716-19.2 TABLE S1 Associations of HIV Env-specific binding antibody responses to a panel of HIV-1 antigens in breast milk and plasma and HIV-1 transmission to the infant. Odds ratios (ORs) and 95% confidence intervals (CIs) were determined by logistic regression modelling controlling for log_10_ plasma viral load. ^a^Immune response is dichotomous, with categories of below the lower limit of detection (LLD) and above the LLD. The odds ratio interprets as above the LLD relative to below the LLD. Download Table S1, DOCX file, 0.02 MB. Copyright © 2019 Hompe et al. 2019 Hompe et al. This content is distributed under the terms of the Creative Commons Attribution 4.0 International license.

However, several plasma and breast milk IgG binding responses were positively associated with postnatal transmission risk prior to correction for multiple comparisons (results for multiple comparisons are shown in Table S1). Plasma IgG responses directed against three HIV Env epitopes—the V3 loop peptide, a consensus clade B gp120 protein, and the V1V2 region from a clade C virus—were positively associated with increased transmission risk (OR = 3.92 [1.04, 14.69] and *P* = 0.04; OR = 10.56 [1.13, 98.26] and *P* = 0.04; OR = 3.36 [1.08, 10.49] and *P* = 0.04). In addition, breast milk IgG directed against the consensus clade B gp120 epitope was similarly associated with increased postnatal transmission risk (OR = 4.49 [1.13, 17.83] and *P* = 0.03).

## DISCUSSION

In this study, we assessed HIV Env-specific binding and functional antibody responses in breast milk and plasma and their association with postnatal HIV-1 transmission risk in women treated with antiretrovirals (ARVs) during pregnancy who received variable postnatal prophylaxis in the IMPAACT PROMISE study. A comparison of Env-specific antibody responses in breast milk versus plasma has not been reported for low-risk transmitting women and has key implications for the development of vaccine interventions to eliminate postnatal HIV transmission. Prior studies have consistently demonstrated that in HIV/simian immunodeficiency virus (SIV) infection, there is an impaired IgA response in breast milk and other mucosal compartments (26–28). While Env-specific IgG and ADCC activity in plasma and breast milk were directly correlated, we previously described the lack of correlation between HIV-1 Env-binding IgA responses in breast milk and plasma in a cohort of HIV-infected women with minimal ART coverage (25). Yet in this cohort, we found that Env-specific IgA binding responses were highly correlated in breast milk and plasma and were comparable in magnitude.

Breast milk B.con gp140-specific total IgA and sIgA responses were inconclusive as to the risk of postnatal transmission in regression models adjusted for maternal viral load, with point estimates inconsistent with previous findings from the BAN study (23). Plasma Env-specific IgA binding responses, plasma and breast milk ADCC activity, and Env epitope-specific binding antibody responses were also not significantly associated with MTCT risk. As our analyses were limited by the small number of transmitting women in this cohort, we are unable to make definitive conclusions about the association of maternal antibody responses with postnatal transmission risk. We found moderate correlations between the magnitudes of plasma IgA, breast milk IgA, and breast milk sIgA binding to B.con gp140 and maternal plasma viral load, suggesting that the high levels of binding antibody responses observed in transmitting, healthy women could be at least partially driven by ongoing virus replication and antigen exposure.

Our analyses of breast milk and plasma maternal ADCC activity revealed no conclusive association with transmission risk and thus contribute to an ongoing debate regarding the potentially protective role of ADCC in postnatal transmission. Prior work has shown that ADCC responses may contribute to better viral control in chronic SIV infection (29, 30) and mediate partial protection in SIV-challenged rhesus monkeys (31). However, several human studies have shown no association between maternal ADCC responses and risk of HIV-1 transmission (23, 32). Milligan et al. evaluated the ADCC activity of antibodies passively transferred from HIV-infected mothers to their infants and found higher levels of ADCC activity in uninfected than in infected infants (32). ADCC activity in infected infants was also associated with decreased mortality, suggesting that it may mediate disease progression and contribute to improved clinical outcomes in HIV infection. Mabuka et al. recently demonstrated higher levels of Env gp120-specific ADCC activity in breast milk in nontransmitting than in transmitting mothers, further implicating ADCC as a potentially protective immune correlate (22). However, this study selected women with high viral loads and systemic neutralizing antibodies. It is possible that maternal ADCC responses are protective only in the setting of high antigen burden, supported by the fact that in our study, one-third of mothers had ADCC titers below the limit of detection. Sun et al. (30) also found an association between ADCC responses and improved viral control in SIV infection when rhesus monkeys were stratified by viral load and those with low viral load excluded.

Our analysis facilitates a comparison to prior studies examining immune parameters that are associated with postnatal transmission risk in different cohorts of transmitting and nontransmitting HIV-infected, breastfeeding women (Table 5). Studies by Mabuka et al., Kuhn et al., and Pollara et al. examined immune correlates in women from the Kenyan Breastfeeding versus Formula Feeding Study, Zambia Exclusive Breastfeeding Study, and BAN trial, respectively (21–23). The cohorts were comprised of clade C-infected women and had comparable sample sizes, with the exception of the study by Mabuka et al., which compared 9 transmitting to 10 nontransmitting women, all clade A infected. The PROMISE cohort included clade C-infected women from Malawi, India, and South Africa, as well as clade A- and D-infected women from Uganda. Women in the PROMISE antepartum (AP) component randomized to maternal triple ART or monotherapy received more ARVs, with better maternal immune status than that of women in the other studies. However, postnatal prophylaxis in the PROMISE study varied, with mothers randomized to receive triple ART for the duration of breastfeeding (42%), to receive 7 to 14 days of ART postpartum (26%), or to the infant nevirapine prophylaxis arm (32%).

**TABLE 5:** Comparison of the PROMISE analysis and prior studies of maternal humoral immune correlates of postnatal transmission in different cohorts of transmitting and nontransmitting HIV-infected breastfeeding women

Parameter	Mabuka et al., 2012 (22)	Kuhn et al., 2006 (21)	Pollara et al., 2015 (23)	Hompe et al., 2019 (this study)
Study cohort	Kenya Breastfeeding vs. Formula Feeding Study	Zambia Exclusive Breastfeeding Study	Malawi Breastfeeding, Antiretrovirals, and Nutrition Study	African multisite Promoting Maternal and Infant Survival Everywhere Study
Sample size	9 transmitting and 10 nontransmitting mothers	26 transmitting and 64 nontransmitting mothers	22 transmitting and 65 nontransmitting mothers	19 transmitting and 57 nontransmitting mothers
Viral clade of infection	Clade A	Clade C	Clade C	Clade C (Malawi, India, South Africa); clade A/D (Uganda)
ART administration	No maternal ART	Single-dose nevirapine	Single-dose nevirapine at onset of labor and then 7 days of zidovudine/lamivudine	Maternal triple ART or infant nevirapine prophylaxis
Maternal immune status	No CD4+ T cell count restriction	No CD4^+^ T cell count restriction	CD4^+^ T cell count >200	CD4^+^ T cell count of >350
Breast milk humoral immune variables and their association with transmission risk				
ADCC activity	Positively associated with nontransmission	Not studied	Not associated with transmission	Not associated with transmission
Env-specific IgA	Not associated with transmission	Not studied	Positively associated with nontransmission	Not associated with transmission
Env-specific sIgA	Not studied	Not associated with transmission	Positively associated with nontransmission	Not associated with transmission

Differences in maternal humoral immune responses associated with postnatal transmission were observed across studies. Mabuka et al. (22) reported that ADCC activity was associated with reduced postnatal transmission risk in a cohort of untreated women, in contrast to the subsequent analyses which have shown no clear association between breast milk ADCC activity and postnatal transmission risk in mothers receiving ART. With regard to HIV Env-specific antibody responses, Kuhn et al. (21) found that sIgA was detected more often in breast milk of 26 postnatal transmitting mothers (76.9%) than in that of 64 nontransmitting mothers (46.9%; *P* = 0.009) and thus did not appear to be protective against MTCT. Pollara et al. (23) identified total IgA and sIgA binding to B.con gp140 by enzyme-linked immunosorbent assay (ELISA) and BAMA, respectively, as being associated with decreased postnatal transmission risk in women who received only peripartum nevirapine, while this analysis of the small number of postnatal infections that occurred in the PROMISE study was inconclusive as to the association of maternal humoral immune responses with postnatal transmission risk. It is possible that previously identified antibody responses are not as robust and do not mediate any protective benefit in the setting of better-controlled viremia due to ARV use throughout pregnancy and immediately postpartum.

While our study is unique in its assessment of maternal antibody responses and transmission risk in the setting of ARV use during pregnancy and postpartum, it had several limitations. The analysis was underpowered, limited by sample size due to the small number of transmissions during breastfeeding. Unfortunately, due to issues with shipping and transportation from study sites in Tanzania and Zimbabwe, only 76 of 104 originally requested samples were available for inclusion in the study. Sample size limited our ability to perform analyses stratified by postpartum ARV status or the type of peripartum ARV regimen. Larger studies using cohorts of ART-treated women should be done to better define the association between maternal binding antibody responses and ADCC activity and postnatal transmission risk. Further study also needs to evaluate the effect of potential confounding factors—adherence and the development of drug resistance—on postnatal transmission risk in the setting of lifetime maternal ART. In addition, another opportunity for future study could include analyzing the data by study site to determine the effect of geographical areas, HIV clade, and coexisting infections on humoral immune responses and postnatal transmission. This could not be done in the current case-control study because study site was a matching criterion.

Interestingly, our work suggests that ARV use during pregnancy significantly alters the nature of breast milk IgA responses, eliminating the deficiency in mucosal IgA typically observed in HIV-infected women (26–28). In women treated with ARVs in pregnancy and at low risk of transmission postpartum due to optimal prophylaxis, antibody responses and viral replication may be strongly correlated and thus antibody responses may not independently affect transmission risk. Our prior observation that maternal humoral immune responses in breast milk are associated with protection against postnatal HIV transmission was not confirmed in this small cohort of low-risk transmitters, yet it merits further study. As highly active ART is the standard of care worldwide, investigating immune correlates of protection in cohorts of well-treated women is critical to the design of strategies to augment infant protection and achieve complete elimination of MTCT.

## MATERIALS AND METHODS

### Study design and participants.

The 1077BF protocol of the IMPAACT PROMISE study enrolled HIV-infected, postpartum women who intended to breastfeed and had CD4^+^ T cell counts of ≥350 cells/mm^3^ between June 2011 and October 2014 (5). Mothers were followed until the trial ended, and their infants were followed through 24 months of age. For this analysis, study participants came from 3 components of the 1077BF protocol, antepartum (AP), postpartum (PP), and late presenters (LP) (Fig. S1).

10.1128/mSphere.00716-19.1 FIG S1 PROMISE 1077BF study components and participants included in the immune correlates analysis. This schema depicts the selection of study participants from the 3 components of the PROMISE 1077BF study (antepartum [AP], postpartum [PP], and late presenters [LP]) and the prophylaxis regimen of each mother. The number of MTCT cases in each component is shown, as are the reasons for exclusion of particular cases from the immune correlates analysis. Overall, there were 19 mothers who transmitted HIV-1 during breastfeeding who met the criteria for this study and had samples available (*n* = 5 from AP component, *n* = 13 from PP component, and *n* = 1 from LP component). They were matched in a 1:3 overall ratio to nontransmitting women (*n* = 5 from AP component and *n* = 52 from PP component). Download FIG S1, TIF file, 1.1 MB. Copyright © 2019 Hompe et al. 2019 Hompe et al. This content is distributed under the terms of the Creative Commons Attribution 4.0 International license.

In the AP component, HIV-infected mothers (*n* = 3,543) were randomized during pregnancy at ≥14 weeks of gestation to receive triple ART (lopinavir-ritonavir [LPV/r], zidovudine [ZDV], and lamivudine or LPV/r plus tenofovir-emtricitabine [TDF/FTC]), or ZDV with a single dose of nevirapine (NVP) and TDF/FTC tail. In the LP component, participants (*n* = 204) were identified as HIV infected in labor or immediately postpartum (up to 5 days). LP participants were assigned to treatments based on their time of registration and previous treatment use. Participants who remained in LP instead of transitioning to PP after delivery were followed for 6 weeks. Finally, both the AP and LP component participants were able to enroll into the PP component if eligible. PP participants were randomized at 6 to 14 days postpartum to maternal ART or infant NVP prophylaxis, continuing through 18 months after delivery or until cessation of breastfeeding or infant HIV-1 infection. Infants in the maternal ART arm also received 6 weeks of NVP. A total of 2,431 mother-infant pairs were enrolled in the PP component (*n* = 1,220 for maternal ART and *n* = 1,211 for infant NVP); 95% were recruited from the AP component and 5% from the LP component.

In our study, cases were defined as children who tested positive for HIV during the breastfeeding period and had a minimum breast milk sample volume required for testing from the transmitting mother prior to infection (*n* = 26). The time of transmission was defined as the first of two consecutive positive infant HIV nucleic acid tests. Twenty-six cases were identified at 9 sites across 6 countries (India [1 site], Malawi [2], South Africa [1], Tanzania [1], Uganda [1], and Zimbabwe [3]). Seven of the 26 transmission cases had unavailable samples. The 19 postnatal transmission cases with available samples were matched to uninfected infants (controls) in a 3:1 ratio for a total of 57 controls. Cases and controls were matched on the following criteria: adequate sample volumes required for planned assays, nontransmitting mothers whose infants were uninfected at the same study week as the transmission occurred in the cases, availability of samples at the same study week or within 3 months prior to the sample time point for the transmitting mother, and clinical site. The maternal prophylaxis regimens during pregnancy and the postpartum period are detailed in Fig. S1.

### Ethics statement.

Approval for the PROMISE 1077BF Protocol was obtained from all local institutional review boards and regulatory authorities. All women who participated in the trial provided written informed consent. The National Institute of Allergy and Infectious Diseases Division of AIDS Multinational Data and Safety Monitoring Board reviewed the study every 6 months. This immune-correlate laboratory analysis of the PROMISE 1077BF study was deemed exempt from human subject review by the Duke University institutional review board (Pro00030437).

### ADCC assay.

Plasma and delipidized breast milk (33) were evaluated for antibody-dependent cell cytotoxicity (ADCC) activity using a luciferase-based assay as previously described (23, 34). CEM.NKR_CCR5_ (35) target cells were infected with an infectious molecular clone virus that encodes the HIV-1 subtype C envelope from the breast milk transmitted/founder virus isolate 4403BMC5 (36) in the NL4-3 isogenic backbone, which contains a *Renilla* luciferase reporter gene (37). The frequency of cells expressing intracellular p24 was used to confirm and monitor infections, and greater than 70% of target cells were p24 positive in all assays. Cryopreserved human peripheral blood mononuclear cells (PBMCs) from an HIV-seronegative donor homozygous for the low-affinity single nucleotide polymorphism variant of Fcγ receptor IIIa (158F) were used as a source of effector cells at an effector-to-target cell ratio of 30:1 (38, 39). Plasma and breast milk samples were tested after 5-fold serial dilutions starting at 1:50 and 1:10, respectively, in duplicate. Percent specific killing was measured after a 6-h incubation at 37°C and 5% CO_2_ and was determined by reduction in luminescence (ViviRen assay; Promega) compared to that of control wells containing target and effector cells in the absence of plasma or breast milk according to the following formula: percent specific killing = [(number of RLU of target and effector well − number of RLU of test well)/number of RLU of target and effector well] × 100. The ADCC endpoint titers were determined by interpolating the dilutions of plasma or breast milk that intersected the positive threshold for killing (20% specific killing) and are reported as reciprocal dilutions.

### BAMA.

HIV-1 Env-specific IgG, IgA, and sIgA responses against a panel of HIV-1 antigens were detected by binding antibody multiplex assay (BAMA), as previously described (33, 40). Carboxylated fluorescent beads (Bio-Rad Laboratories, Inc.) coupled with HIV-1 antigens (Table S2) were incubated with diluted plasma or breast milk for 30 min at 20°C. Plasma was IgG depleted prior to measuring Env-specific IgA and sIgA, as previously described (25, 33). HIV Env-specific IgG and IgA were detected with phycoerythrin (PE)-conjugated mouse anti-human IgG (Southern Biotech) and PE-conjugated goat anti-human IgA (Jackson ImmunoResearch Laboratories), respectively. Env-specific sIgA was detected with mouse anti-human secretory component (Sigma-Aldrich) followed by goat anti-mouse IgG-PE (Southern Biotech). Beads were washed and acquired on a Bio-Plex 200 system (Bio-Rad Laboratories, Inc.).

10.1128/mSphere.00716-19.3 TABLE S2 Amino acid sequences and sample dilutions for antigens used in BAMA assays. Download Table S2, DOCX file, 0.02 MB. Copyright © 2019 Hompe et al. 2019 Hompe et al. This content is distributed under the terms of the Creative Commons Attribution 4.0 International license.

To determine the optimal sample dilution for each antigen, optimization assays with serial dilutions of a subset of plasma and breast milk samples were performed. All samples were analyzed at the same dilution for each antigen (Table S2). Blank beads were used to account for nonspecific binding, and HIV immunoglobulin (HIVIG) was used as a positive control. Mean fluorescence intensity (MFI) values were background adjusted by subtracting the MFI values of coupled beads without sample. For IgA assays, MFI values were also blank-bead subtracted to account for nonspecific binding at the low sample dilutions used. A positive HIV-1 Env-specific antibody response was an MFI of >100. All assays tracked the 50% effective concentration and maximum MFI of HIVIG by Levey-Jennings charts to ensure interassay consistency.

### ELISA.

For enzyme-linked immunosorbent assay (ELISA), 384-well plates (Corning Life Sciences) were coated at 30 ng/well with B.con env03 gp140, incubated at 4°C overnight, and blocked with phosphate-buffered saline (PBS) containing 4% whey protein, 15% normal goat serum, and 0.5% Tween 20. Serially diluted breast milk or IgG-depleted plasma samples were added. IgA was detected with goat anti-human IgA horseradish peroxidase (HRP; Southern Biotech), and sIgA was detected with mouse anti-human secretory component (Sigma-Aldrich), followed by goat anti-mouse IgG HRP (Southern Biotech). Plates were developed with SureBlue Reserve substrate and stop solution (VWR) and read at 450 nm on a SpectraMax Plus 384 microplate reader (Molecular Devices). The standard for IgA measurement was VRC01 monomeric IgA. The standard for sIgA assays was CH31 sIgA, prepared by complexing CH31 dimeric IgA with human secretory component (Abcam) in a 1:1 molar ratio (41). A five-parameter curve was used to calculate IgA/sIgA concentrations relative to the respective standard (SoftMax Pro 6.3; Molecular Devices).

### Statistical analysis.

Maternal and infant characteristics were summarized by case-control status using the minimum, maximum, mean (standard deviation [SD]), and median (Q1, Q3) for continuous variables and number (percent) for categorical variables (Table 1). The distribution of values for breast milk and plasma immune responses were summarized by the minimum, maximum, mean (SD), and median (Q1, Q3) (Table S3). If any immune response had >30% of the values below the lower limit of detection (LLD), a dichotomous measure of the immune response was used, with categories of below LLD and above LLD (Table S3). Correlations were investigated with Spearman correlation coefficients, and their respective *P* values, for cases and controls combined, between each immune response in plasma and breast milk, between immune responses and maternal plasma viral load, and between ADCC potency and plasma Env-specific IgG. Conditional logistic regression models were fit to estimate the odds of postnatal HIV-1 transmission for a 1-unit change in breast milk and plasma immune responses as measured by the ADCC assay, ELISA, and BAMA (Table 6). Odds ratios and 95% confidence intervals were generated. Adjusted logistic regression models were fit separately with each of the following variables identified *a priori* as potential confounders: maternal viral load (at baseline and closest measure to breast milk sample collection), maternal peripheral CD4^+^ T cell count (at baseline, and closest measure to breast milk sample collection), AP/PP component randomization, infant age at sample collection, and maternal parity. A potential confounder was included in the final model if it was associated with the outcome and altered the effect estimates, which was only the case for maternal plasma viral load.

**TABLE 6:** Primary and secondary immune variables used in conditional logistic regression models to assess associations with postnatal HIV-1 transmission

Variable type	Immune response	Assay
Primary immune variables	Breast milk total IgA against HIV-1 B.con env03 gp140, measured by AUC	ELISA
	Breast milk sIgA against HIV-1 B.con env03 gp140, measured by AUC	ELISA
	Breast milk ADCC antibody titer (above and below detection)	ADCC-Luc
Secondary immune variables	Plasma total IgA against HIV-1 B.con env03 gp140, measured by AUC	ELISA
	Plasma ADCC antibody titer (above and below detection)	ADCC-Luc
	Breast milk ADCC potency (maximum % specific killing)	ADCC-Luc
	Plasma ADCC potency (maximum % specific killing)	ADCC-Luc
	Plasma Env-specific IgG (against a panel of HIV-1 antigens)	BAMA
	Breast milk Env-specific IgG (against a panel of HIV-1 antigens)	BAMA
	Breast milk total IgA (against a panel of HIV-1 antigens)	BAMA
	Plasma total IgA (against a panel of HIV-1 antigens)	BAMA
	Breast milk sIgA (against a panel of HIV-1 antigens)	BAMA

10.1128/mSphere.00716-19.4 TABLE S3 Distribution of values for breast milk and plasma immune responses. ^a^Immune response is dichotomous, with categories of below the LLD and above the LLD. Download Table S3, DOCX file, 0.01 MB. Copyright © 2019 Hompe et al. 2019 Hompe et al. This content is distributed under the terms of the Creative Commons Attribution 4.0 International license.

## References

[1] Thomas TK, Masaba R, Borkowf CB, Ndivo R, Zeh C, Misore A, Otieno J, Jamieson D, Thigpen MC, Bulterys M, Slutsker L, De Cock KM, Amornkul PN, Greenberg AE, Fowler MG, KiBS Study Team. 2011 Triple-antiretroviral prophylaxis to prevent mother-to-child HIV transmission through breastfeeding—the Kisumu Breastfeeding Study. PLoS Med 8:e1001015. doi:10.1371/journal.pmed.1001015. 21468300 PMC3066129

[2] Kilewo C, Karlsson K, Massawe A, Lyamuya E, Swai A, Mhalu F, Biberfeld G, Mitra Study Team. 2008 Prevention of mother-to-child transmission of HIV-1 through breast-feeding by treating infants prophylactically with lamivudine in Dar es Salaam, Tanzania: the Mitra study. J Acquir Immune Defic Syndr 48:315–323. doi:10.1097/QAI.0b013e31816e395c. 18344879

[3] Palombi L, Marazzi MC, Voetberg A, Magid NA, the DREAM Program Prevention of Mother-To-Child Transmission Team. 2007 Treatment acceleration program and the experience of the DREAM program in prevention of mother-to-child transmission of HIV. AIDS 21:S65–S71. doi:10.1097/01.aids.0000279708.09180.f5. 17620755

[4] Chasela CS, Hudgens MG, Jamieson DJ, Kayira D, Hosseinipour MC, Kourtis AP, Martinson F, Tegha G, Knight RJ, Ahmed YI, Kamwendo DD, Hoffman IF, Ellington SR, Kacheche Z, Soko A, Wiener JB, Fiscus SA, Kazembe P, Mofolo IA, Chigwenembe M, Sichali DS, van der Horst CM 2010 Maternal or infant antiretroviral drugs to reduce HIV-1 transmission. N Engl J Med 362:2271–2281. doi:10.1056/NEJMoa0911486. 20554982 PMC3440865

[5] Flynn PM, Taha TE, Cababasay M, Fowler MG, Mofenson LM, Owor M, Fiscus S, Stranix-Chibanda L, Coutsoudis A, Gnanashanmugam D, Chakhtoura N, McCarthy K, Mukuzunga C, Makanani B, Moodley D, Nematadzira T, Kusakara B, Patil S, Vhembo T, Bobat R, Mmbaga BT, Masenya M, Nyati M, Theron G, Mulenga H, Butler K, Shapiro DE, PROMISE Study Team. 2018 Prevention of HIV-1 transmission through breastfeeding: efficacy and safety of maternal antiretroviral therapy versus infant nevirapine prophylaxis for duration of breastfeeding in HIV-1-infected women with high CD4 cell count (IMPAACT PROMISE): a randomized, open-label, clinical trial. J Acquir Immune Defic Syndr 77:383–392. doi:10.1097/QAI.0000000000001612. 29239901 PMC5825265

[6] UNAIDS. 2016 On the fast-track to an AIDS-free generation: the incredible journey of the global plan towards the elimination of new HIV infections among children by 2015 and keeping their mothers alive. UNAIDS, Geneva, Switzerland.

[7] Coutsoudis A, Dabis F, Fawzi W, Gaillard P, Haverkamp G, Harris DR, Jackson JB, Leroy V, Meda N, Msellati P, Newell M-L, Nsuati R, Read JS, Wiktor S 2004 Late postnatal transmission of HIV-1 in breast-fed children: an individual patient data meta-analysis. J Infect Dis 189:2154–2166. 15181561 10.1086/420834

[8] John-Stewart G, Mbori-Ngacha D, Ekpini R, Janoff EN, Nkengasong J, Read JS, Van de Perre P, Newell M-L 2004 Breast-feeding and transmission of HIV-1. J Acquir Immune Defic Syndr 35:196–202. doi:10.1097/00126334-200402010-00015. 14722454 PMC3382106

[9] Semba RD, Kumwenda N, Hoover DR, Taha TE, Quinn TC, Mtimavalye L, Biggar RJ, Broadhead R, Miotti PG, Sokoll LJ, van der Hoeven L, Chiphangwi JD 1999 Human immunodeficiency virus load in breast milk, mastitis, and mother-to-child transmission of human immunodeficiency virus type 1. J Infect Dis 180:93–98. doi:10.1086/314854. 10353866

[10] John GC, Nduati RW, Mbori-Ngacha DA, Richardson BA, Panteleeff D, Mwatha A, Overbaugh J, Bwayo J, Ndinya-Achola JO, Kreiss JK 2001 Correlates of mother-to-child human immunodeficiency virus type 1 (HIV-1) transmission: association with maternal plasma HIV-1 RNA load, genital HIV-1 DNA shedding, and breast infections. J Infect Dis 183:206–212. doi:10.1086/317918. 11120927

[11] Creek TL, Kim A, Lu L, Bowen A, Masunge J, Arvelo W, Smit M, Mach O, Legwaila K, Motswere C, Zaks L, Finkbeiner T, Povinelli L, Maruping M, Ngwaru G, Tebele G, Bopp C, Puhr N, Johnston SP, Dasilva AJ, Bern C, Beard RS, Davis MK 2010 Hospitalization and mortality among primarily nonbreastfed children during a large outbreak of diarrhea and malnutrition in Botswana, 2006. J Acquir Immune Defic Syndr 53:14–19. doi:10.1097/QAI.0b013e3181bdf676. 19801943

[12] Shapiro RL, Lockman S, Kim S, Smeaton L, Rahkola JT, Thior I, Wester C, Moffat C, Arimi P, Ndase P, Asmelash A, Stevens L, Montano M, Makhema J, Essex M, Janoff EN 2007 Infant morbidity, mortality, and breast milk immunologic profiles among breast-feeding HIV-infected and HIV-uninfected women in Botswana. J Infect Dis 196:562–569. doi:10.1086/519847. 17624842

[13] Kuhn L, Aldrovandi GM, Sinkala M, Kankasa C, Semrau K, Mwiya M, Kasonde P, Scott N, Vwalika C, Walter J, Bulterys M, Tsai WY, Thea DM, Zambia Exclusive Breastfeeding Study. 2008 Effects of early, abrupt weaning on HIV-free survival of children in Zambia. N Engl J Med 359:130–141. doi:10.1056/NEJMoa073788. 18525036 PMC2577610

[14] Iliff PJ, Piwoz EG, Tavengwa NV, Zunguza CD, Marinda ET, Nathoo KJ, Moulton LH, Ward BJ, Humphrey JH, ZVITAMBO study group. 2005 Early exclusive breastfeeding reduces the risk of postnatal HIV-1 transmission and increases HIV-free survival. AIDS 19:699–708. doi:10.1097/01.aids.0000166093.16446.c9. 15821396

[15] Coovadia HM, Rollins NC, Bland RM, Little K, Coutsoudis A, Bennish ML, Newell ML 2007 Mother-to-child transmission of HIV-1 infection during exclusive breastfeeding in the first 6 months of life: an intervention cohort study. Lancet 369:1107–1116. doi:10.1016/S0140-6736(07)60283-9. 17398310

[16] World Health Organization. 2016 Guideline: updates on HIV and infant feeding: the duration of breastfeeding, and support from health services to improve feeding practices among mothers living with HIV. World Health Organization, Geneva, Switzerland. 27583316

[17] Nduati R, John G, Mbori-Ngacha D, Richardson B, Overbaugh J, Mwatha A, Ndinya-Achola J, Bwayo J, Onyango FE, Hughes J, Kreiss J 2000 Effect of breastfeeding and formula feeding on transmission of HIV-1: a randomized clinical trial. JAMA 283:1167–1174. doi:10.1001/jama.283.9.1167. 10703779

[18] De Cock KM, Fowler M, Mercier E, de Vincenzi I, Saba J, Hoff E, Alnwick DJ, Rogers M, Shaffer N 2000 Prevention of mother-to-child HIV transmission in resource-poor countries: translating research into policy and practice. JAMA 283:1175–1182. doi:10.1001/jama.283.9.1175. 10703780

[19] Duprat C, Mohammed Z, Datta P, Stackiw W, Ndinya-Achola JO, Kreiss JK, Holmes KK, Plummer FA, Embree JE 1994 Human immunodeficiency virus type 1 IgA antibody in breast milk and serum. Pediatr Infect Dis J 13:603–608. doi:10.1097/00006454-199407000-00004. 7970947

[20] Becquart P, Chomont N, Roques P, Ayouba A, Kazatchkine MD, Belec L, Hocini H 2002 Compartmentalization of HIV-1 between breast milk and blood of HIV-infected mothers. Virology 300:109–117. doi:10.1006/viro.2002.1537. 12202211

[21] Kuhn L, Trabattoni D, Kankasa C, Sinkala M, Lissoni F, Ghosh M, Aldrovandi G, Thea D, Clerici M 2006 HIV-specific secretory IgA in breast milk of HIV-positive mothers is not associated with protection against HIV transmission among breast-fed infants. J Pediatr 149:611–616. doi:10.1016/j.jpeds.2006.06.017. 17095329 PMC2811256

[22] Mabuka J, Nduati R, Odem-Davis K, Peterson D, Overbaugh J 2012 HIV-specific antibodies capable of ADCC are common in breastmilk and are associated with reduced risk of transmission in women with high viral loads. PLoS Pathog 8:e1002739. doi:10.1371/journal.ppat.1002739. 22719248 PMC3375288

[23] Pollara J, McGuire E, Fouda GG, Rountree W, Eudailey J, Overman RG, Seaton KE, Deal A, Edwards RW, Tegha G, Kamwendo D, Kumwenda J, Nelson JAE, Liao H-X, Brinkley C, Denny TN, Ochsenbauer C, Ellington S, King CC, Jamieson DJ, van der Horst C, Kourtis AP, Tomaras GD, Ferrari G, Permar SR 2015 Association of HIV-1 envelope-specific breast milk IgA responses with reduced risk of postnatal mother-to-child transmission of HIV-1. J Virol 89:9952–9961. doi:10.1128/JVI.01560-15. 26202232 PMC4577885

[24] Fowler MG, Qin M, Fiscus SA, Currier JS, Flynn PM, Chipato T, McIntyre J, Gnanashanmugam D, Siberry GK, Coletti AS, Taha TE, Klingman KL, Martinson FE, Owor M, Violari A, Moodley D, Theron GB, Bhosale R, Bobat R, Chi BH, Strehlau R, Mlay P, Loftis AJ, Browning R, Fenton T, Purdue L, Basar M, Shapiro DE, Mofenson LM 2016 Benefits and risks of antiretroviral therapy for perinatal HIV prevention. N Engl J Med 375:1726–1737. doi:10.1056/NEJMoa1511691. 27806243 PMC5214343

[25] Fouda GG, Yates NL, Pollara J, Shen X, Overman GR, Mahlokozera T, Wilks AB, Kang HH, Salazar-Gonzalez JF, Salazar MG, Kalilani L, Meshnick SR, Hahn BH, Shaw GM, Lovingood RV, Denny TN, Haynes B, Letvin NL, Ferrari G, Montefiori DC, Tomaras GD, Permar SR, Center for HIV/AIDS Vaccine Immunology. 2011 HIV-specific functional antibody responses in breast milk mirror those in plasma and are primarily mediated by IgG antibodies. J Virol 85:9555–9567. doi:10.1128/JVI.05174-11. 21734046 PMC3165739

[26] Rychert J, Amedee AM 2005 The antibody response to SIV in lactating rhesus macaques. J Acquir Immune Defic Syndr 38:135–141. doi:10.1097/01.qai.0000148947.03416.b5. 15671797

[27] Becquart P, Hocini H, Levy M, Sepou A, Kazatchkine MD, Belec L 2000 Secretory anti-human immunodeficiency virus (HIV) antibodies in colostrum and breast milk are not a major determinant of the protection of early postnatal transmission of HIV. J Infect Dis 181:532–539. doi:10.1086/315255. 10669336

[28] Permar SR, Wilks AB, Ehlinger EP, Kang HH, Mahlokozera T, Coffey RT, Carville A, Letvin NL, Seaman MS 2010 Limited contribution of mucosal IgA to simian immunodeficiency virus (SIV)-specific neutralizing antibody response and virus envelope evolution in breast milk of SIV-infected, lactating rhesus monkeys. J Virol 84:8209–8218. doi:10.1128/JVI.00656-10. 20519381 PMC2916548

[29] Banks ND, Kinsey N, Clements J, Hildreth JE 2002 Sustained antibody-dependent cell-mediated cytotoxicity (ADCC) in SIV-infected macaques correlates with delayed progression to AIDS. AIDS Res Hum Retroviruses 18:1197–1205. doi:10.1089/08892220260387940. 12487826

[30] Sun Y, Asmal M, Lane S, Permar SR, Schmidt SD, Mascola JR, Letvin NL 2011 Antibody-dependent cell-mediated cytotoxicity in simian immunodeficiency virus-infected rhesus monkeys. J Virol 85:6906–6912. doi:10.1128/JVI.00326-11. 21593181 PMC3126600

[31] Barouch DH, Liu J, Li H, Maxfield LF, Abbink P, Lynch DM, Iampietro MJ, SanMiguel A, Seaman MS, Ferrari G, Forthal DN, Ourmanov I, Hirsch VM, Carville A, Mansfield KG, Stablein D, Pau MG, Schuitemaker H, Sadoff JC, Billings EA, Rao M, Robb ML, Kim JH, Marovich MA, Goudsmit J, Michael NL 2012 Vaccine protection against acquisition of neutralization-resistant SIV challenges in rhesus monkeys. Nature 482:89–93. doi:10.1038/nature10766. 22217938 PMC3271177

[32] Milligan C, Richardson BA, John-Stewart G, Nduati R, Overbaugh J 2015 Passively acquired antibody-dependent cellular cytotoxicity (ADCC) activity in HIV-infected infants is associated with reduced mortality. Cell Host Microbe 17:500–506. doi:10.1016/j.chom.2015.03.002. 25856755 PMC4392343

[33] Eudailey JA, Dennis ML, Parker ME, Phillips BL, Huffman TN, Bay CP, Hudgens MG, Wiseman RW, Pollara JJ, Fouda GG, Ferrari G, Pickup DJ, Kozlowski PA, Van Rompay KKA, De Paris K, Permar SR 2018 Maternal HIV-1 Env vaccination for systemic and breast milk immunity to prevent oral SHIV acquisition in infant macaques. mSphere 3:e00505-17. doi:10.1128/mSphere.00505-17. 29359183 PMC5760748

[34] Pollara J, Orlandi C, Beck C, Edwards RW, Hu Y, Liu S, Wang S, Koup RA, Denny TN, Lu S, Tomaras GD, DeVico A, Lewis GK, Ferrari G 2018 Application of area scaling analysis to identify natural killer cell and monocyte involvement in the GranToxiLux antibody dependent cell-mediated cytotoxicity assay. Cytometry A 93:436–447. doi:10.1002/cyto.a.23348. 29498807 PMC5969088

[35] Trkola A, Matthews J, Gordon C, Ketas T, Moore JP 1999 A cell line-based neutralization assay for primary human immunodeficiency virus type 1 isolates that use either the CCR5 or the CXCR4 coreceptor. J Virol 73:8966–8974. 10516002 10.1128/jvi.73.11.8966-8974.1999 PMC112928

[36] Fouda GG, Mahlokozera T, Salazar-Gonzalez JF, Salazar MG, Learn G, Kumar SB, Dennison SM, Russell E, Rizzolo K, Jaeger F, Cai F, Vandergrift NA, Gao F, Hahn B, Shaw GM, Ochsenbauer C, Swanstrom R, Meshnick S, Mwapasa V, Kalilani L, Fiscus S, Montefiori D, Haynes B, Kwiek J, Alam SM, Permar SR 2013 Postnatally-transmitted HIV-1 Envelope variants have similar neutralization-sensitivity and function to that of nontransmitted breast milk variants. Retrovirology 10:3. doi:10.1186/1742-4690-10-3. 23305422 PMC3564832

[37] Edmonds TG, Ding H, Yuan X, Wei Q, Smith KS, Conway JA, Wieczorek L, Brown B, Polonis V, West JT, Montefiori DC, Kappes JC, Ochsenbauer C 2010 Replication competent molecular clones of HIV-1 expressing Renilla luciferase facilitate the analysis of antibody inhibition in PBMC. Virology 408:1–13. doi:10.1016/j.virol.2010.08.028. 20863545 PMC2993081

[38] Koene HR, Kleijer M, Algra J, Roos D, von Dem Borne AE, de Haas M 1997 Fc gammaRIIIa-158V/F polymorphism influences the binding of IgG by natural killer cell Fc gammaRIIIa, independently of the Fc gammaRIIIa-48L/R/H phenotype. Blood 90:1109–1114. doi:10.1182/blood.V90.3.1109. 9242542

[39] Bruhns P, Iannascoli B, England P, Mancardi DA, Fernandez N, Jorieux S, Daeron M 2009 Specificity and affinity of human Fcgamma receptors and their polymorphic variants for human IgG subclasses. Blood 113:3716–3725. doi:10.1182/blood-2008-09-179754. 19018092

[40] Tomaras GD, Yates NL, Liu P, Qin L, Fouda GG, Chavez LL, Decamp AC, Parks RJ, Ashley VC, Lucas JT, Cohen M, Eron J, Hicks CB, Liao H-X, Self SG, Landucci G, Forthal DN, Weinhold KJ, Keele BF, Hahn BH, Greenberg ML, Morris L, Karim SSA, Blattner WA, Montefiori DC, Shaw GM, Perelson AS, Haynes BF 2008 Initial B-cell responses to transmitted human immunodeficiency virus type 1: virion-binding immunoglobulin M (IgM) and IgG antibodies followed by plasma anti-gp41 antibodies with ineffective control of initial viremia. J Virol 82:12449–12463. doi:10.1128/JVI.01708-08. 18842730 PMC2593361

[41] Nelson CS, Pollara J, Kunz EL, Jeffries TL Jr, Duffy R, Beck C, Stamper L, Wang M, Shen X, Pickup DJ, Staats HF, Hudgens MG, Kepler TB, Montefiori DC, Moody MA, Tomaras GD, Liao HX, Haynes BF, Ferrari G, Fouda GGA, Permar SR 2016 Combined HIV-1 envelope systemic and mucosal immunization of lactating rhesus monkeys induces a robust immunoglobulin A isotype B cell response in breast milk. J Virol 90:4951–4965. doi:10.1128/JVI.00335-16. 26937027 PMC4859715

