# Supplementary material for: Maternal Humoral Immune Responses Do Not Predict Postnatal HIV-1 Transmission Risk in Antiretroviral-Treated Mothers from the IMPAACT PROMISE Study
Source: mSphere. 2019 Oct 23;4(5):e00716-19. doi: 10.1128/mSphere.00716-19 (PMC7407004; doi:10.1128/mSphere.00716-19)
Supplement: TABLE S1 [file mSphere.00716-19-st001.docx]

| **Antibody Response** | **Analyte** | **Odds Ratio (95% CI)** | **P-value^a^** | **False Discover Rate (FDR) p-value** |
| --- | --- | --- | --- | --- |
| Plasma IgG | Bio-V3.C | 3.92 (1.04, 14.69) | 0.04 | 0.13 |
|  | 4403 BMC5 gp120 | 21.76 (0.89, 529.38) | 0.06 | 0.13 |
|  | 1209 BMH5 gp120 | 21.82 (0.92, 515.71) | 0.06 | 0.13 |
|  | Con6 gp120/B | 10.56 (1.13, 98.26) | 0.04 | 0.13 |
|  | ConS gp140 | 6.08 (0.46, 80.15) | 0.17 | 0.20 |
|  | 1086C gp140 | 17.85 (0.64, 496.77) | 0.09 | 0.13 |
|  | Rec MN gp41 | 1.28 (0.37, 4.40) | 0.69 | 0.69 |
|  | gp70 V1V2 1086.C | 3.36 (1.08, 10.49) | 0.04 | 0.13 |
|  | gp70 B.caseA V1V2 | 2.35 (0.89, 6.23) | 0.09 | 0.13 |
|  | gp70 conC V3 tags | 3.75 (0.59, 23.71) | 0.16 | 0.20 |
|  | A1.con env03 gp140 | 8.69 (0.75, 100.48) | 0.08 | 0.13 |
|  | B.con_env03 gp140 | 3.69 (0.50, 27.20) | 0.20 | 0.22 |
|  |  |  |  |  |
| Breast milk IgG | Bio-V3.C | 2.68 (0.93, 7.72) | 0.07 | 0.16 |
|  | 4403 BMC5 gp120 | 5.04 (0.96, 26.42) | 0.06 | 0.16 |
|  | 1209 BMH5 gp120 | 5.05 (0.96, 26.70) | 0.06 | 0.16 |
|  | Con6 gp120/B | 4.49 (1.13, 17.83) | 0.03 | 0.16 |
|  | ConS gp140 | 2.47 (0.63, 9.66) | 0.19 | 0.26 |
|  | 1086C gp140 | 4.55 (0.78, 26.70) | 0.09 | 0.16 |
|  | Rec MN gp41 | 1.36 (0.49, 3.79) | 0.56 | 0.56 |
|  | gp70 V1V2 1086.C | 1.68 (0.93, 3.05) | 0.09 | 0.16 |
|  | gp70 B.caseA V1V2 | 1.29 (0.79, 2.10) | 0.30 | 0.33 |
|  | gp70 conC V3 tags | 2.46 (0.91, 6.63) | 0.08 | 0.16 |
|  | A1.con env03 gp140 | 6.26 (0.69, 56.92) | 0.10 | 0.16 |
|  | B.con_env03 gp140 | 2.70 (0.53, 13.81) | 0.23 | 0.28 |
|  |  |  |  |  |
| Breast milk total IgA | Bio-V3.C | 0.95 (0.36, 2.47) | 0.91 | 0.91 |
|  | 4403 BMC5 gp120^a^ | 1.55 (0.44, 5.47) | 0.50 | 0.83 |
|  | 1209 BMH5 gp120^a^ | 1.56 (0.45, 5.38) | 0.48 | 0.83 |
|  | Con6 gp120/B^a^ | 2.12 (0.46, 9.86) | 0.34 | 0.83 |
|  | ConS gp140^a^ | 1.33 (0.40, 4.44) | 0.64 | 0.83 |
|  | 1086C gp140^a^ | 0.67 (0.17, 2.65) | 0.57 | 0.83 |
|  | Rec MN gp41^a^ | 0.52 (0.11, 2.58) | 0.42 | 0.83 |
|  | gp70 V1V2 1086.C^a^ | 3.35 (0.53, 21.23) | 0.20 | 0.83 |
|  | gp70 B.caseA V1V2^a^ | 2.03 (0.26, 15.86) | 0.50 | 0.83 |
|  | gp70 conC V3 tags^a^ | 0.86 (0.23, 3.18) | 0.82 | 0.90 |
|  | A1.con env03 gp140^a^ | 0.67 (0.16, 2.84) | 0.58 | 0.83 |
|  | B.con_env03 gp140^a^ | 1.29 (0.36, 4.67) | 0.70 | 0.83 |
|  |  |  |  |  |
| Plasma total IgA | Bio-V3.C | 0.77 (0.25, 2.35) | 0.65 | 0.94 |
|  | 4403 BMC5 gp120^a^ | 0.93 (0.23, 3.80) | 0.92 | 0.94 |
|  | 1209 BMH5 gp120^a^ | 0.77 (0.18, 3.37) | 0.73 | 0.94 |
|  | Con6 gp120/B | 1.26 (0.75, 2.13) | 0.39 | 0.94 |
|  | ConS gp140 | 1.07 (0.60, 1.90) | 0.81 | 0.94 |
|  | 1086C gp140 | 0.79 (0.42, 1.49) | 0.46 | 0.94 |
|  | Rec MN gp41 | 1.03 (0.55, 1.94) | 0.92 | 0.94 |
|  | gp70 V1V2 1086.C^a^ | 1.58 (0.25, 10.00) | 0.63 | 0.94 |
|  | gp70 B.caseA V1V2^a^ | 0.80 (0.12, 5.10) | 0.81 | 0.94 |
|  | gp70 conC V3 tags^a^ | 1.05 (0.32, 3.46) | 0.94 | 0.94 |
|  | A1.con env03 gp140^a^ | 0.65 (0.19, 2.18) | 0.48 | 0.94 |
|  | B.con_env03 gp140^a^ | 0.53 (0.15, 1.91) | 0.33 | 0.94 |
|  |  |  |  |  |
| Breast milk sIgA | Bio-V3.C | 1.40 (0.19, 10.14) | 0.74 | 0.98 |
|  | 4403 BMC5 gp120^a^ | 0.99 (0.30, 3.27) | 0.99 | 0.99 |
|  | 1209 BMH5 gp120^a^ | 1.03 (0.31, 3.45) | 0.97 | 0.99 |
|  | Con6 gp120/B^a^ | 2.54 (0.61, 10.64) | 0.20 | 0.52 |
|  | ConS gp140 | 1.87 (0.96, 3.63) | 0.06 | 0.52 |
|  | 1086C gp140^a^ | 1.08 (0.29, 4.06) | 0.91 | 0.99 |
|  | Rec MN gp41 | 0.81 (0.26, 2.51) | 0.71 | 0.98 |
|  | gp70 V1V2 1086.C^a^ | 3.56 (0.53, 23.81) | 0.19 | 0.52 |
|  | gp70 B.caseA V1V2^a^ | 5.20 (0.67, 40.07) | 0.11 | 0.52 |
|  | gp70 conC V3 tags^a^ | 1.49 (0.39, 5.64) | 0.56 | 0.96 |
|  | A1.con env03 gp140^a^ | 1.75 (0.49, 6.30) | 0.39 | 0.78 |
|  | B.con_env03 gp140^a^ | 2.20 (0.63, 7.68) | 0.22 | 0.52 |
|  |  |  |  |  |
