# Supplementary figures and images for: Maternal Humoral Immune Responses Do Not Predict Postnatal HIV-1 Transmission Risk in Antiretroviral-Treated Mothers from the IMPAACT PROMISE Study
Source: mSphere. 2019 Oct 23;4(5):e00716-19. doi: 10.1128/mSphere.00716-19 (PMC7407004; doi:10.1128/mSphere.00716-19)

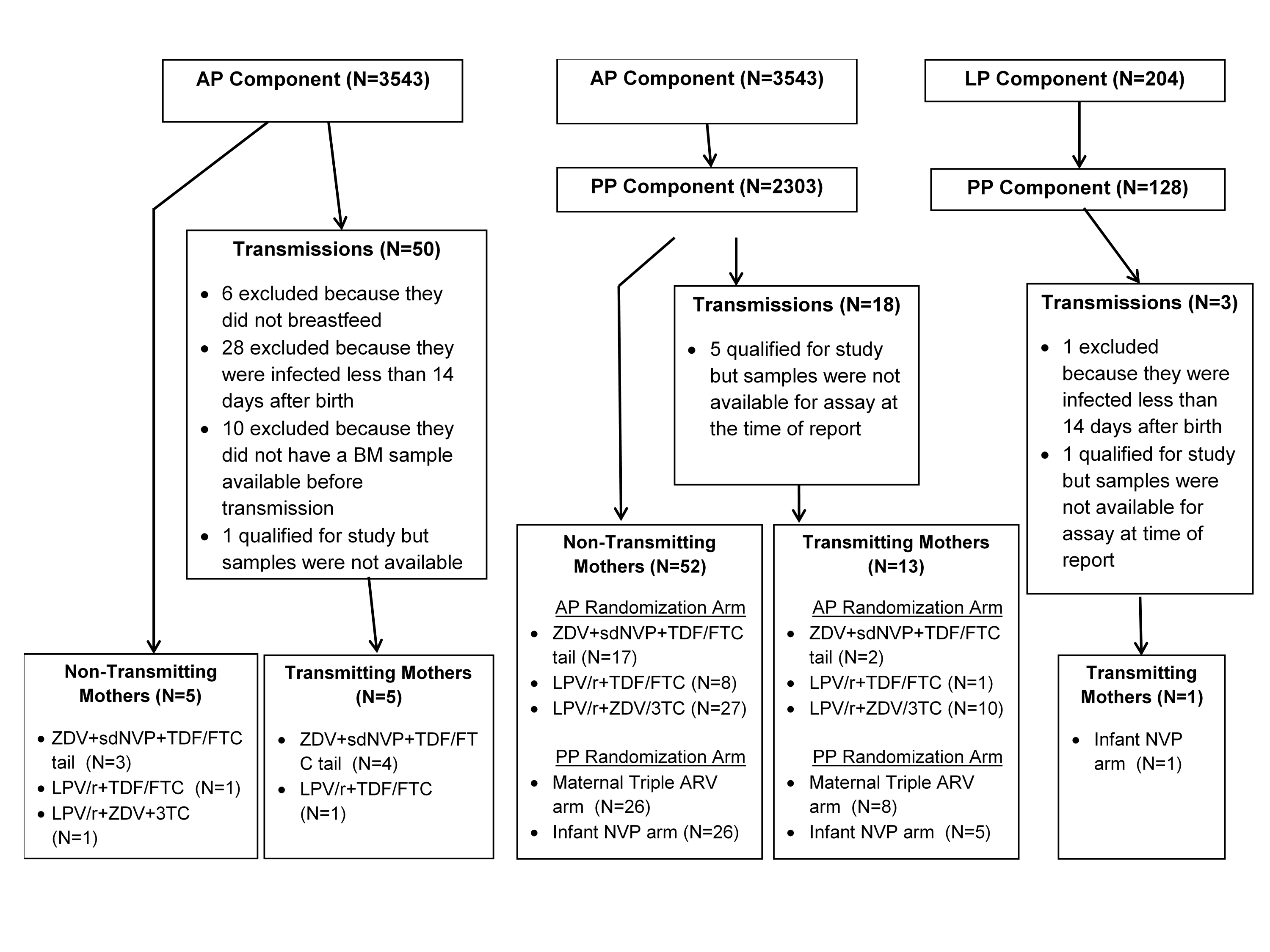

Supplement: FIG S1 [file mSphere.00716-19-sf001.tif]
