# Supplementary material for: Maternal Humoral Immune Responses Do Not Predict Postnatal HIV-1 Transmission Risk in Antiretroviral-Treated Mothers from the IMPAACT PROMISE Study
Source: mSphere. 2019 Oct 23;4(5):e00716-19. doi: 10.1128/mSphere.00716-19 (PMC7407004; doi:10.1128/mSphere.00716-19)
Supplement: TABLE S2 [file mSphere.00716-19-st002.docx]

| **Protein or Peptide** | **Sequence** | **Sample Dilution** | | | | |
| --- | --- | --- | --- | --- | --- | --- |
|  |  | **Plasma** | | **Breast Milk** | | |
|  |  | **IgG** | **IgA** | **IgG** | **IgA** | **sIgA** |
| 4403 BMC5 gp120 | NLWVTVYYGVPVWKEAKTTLFCASDAKAYEREMHNVWATHACVPTDPNPQELVLENVTENFNMWKNDMVDQMHEDIISLWDQSLKPCVKLTPLCVTLDCKNSTENITDSRNITDNGERKNCSFNITTEIKDKKQNVYALFYKLDIVPLHEDNSSYYRLINCNTSAITQACPKVSFDPIPIHYCAPAGYAILKCNNKTFNGTGPCHNVSTVQCTHGIKPVVSTQLLLNGSLAEEEIIIRSKDLTDNAKIIIVHLNKSVEINCTRPGNNTRKSIRIGPGQTFYAPNGIIGNIRKAHCNIKGKQWNETLIKVGEKLAELFPNKIIKFNSSSGGDLEITTHSFNCGGEFFYCDTSGLFNGTYNPNDTTSNSSSLNNITLTLPCKIKQVINMWQGVGKAIYAPPIAGNITCRSNITGLLLLRDGGTTNDTEEIFRPGGGDMRDNWRSELYKYKVVEIKPLGVAPTKAKRRVVEREKRAVGIGAVLLGFLGAAGSTMGAASITLTVQARQLLSGIVQQQSNLLRAIEAQQHMLQLTVWGIKQLQARVLAIERYLKDQQLLGIWGCSGKLICTTSVPWNDSWSHNKSLQDIWDNMTWMQWDKEINNYTSTIYRLLEESQNQQEKNEQDLLALDSWQNLWNWFSISKWLWYIKIFIMIVGGLIGLRIIFAVLSIVNRVRQGYSPLSFQTLTPSPRGPDRLGRIEEEGGEQDRDRSIRLVSGFLALAWDDLRSLCLFLYHRLRDFLLVIARTVEILGRSSLRGLQRGWEALKYLGNLVQYWGSEIKKSAINLFDTIAIAVAEGTDRIIEIIQRIYRAICNIPVRIRQGFEAALL | 1:250 | 1:10 | 1:5 | 1:5 | 1:5 |
| 1209 BMH5 gp120 | NLWVTVYYGVPVWREAKTTLFCASDAKAYDKEVRNVWATHACVPTDPNPQEMILENVTENFNMWKNDMVDQMHEDIISLWDQSLKPCVKLTPLCVTLNCSNVNITDISEAMKEEIRNCSFNITTEIKDKKRKENALFYGLDIVPLDNSGNSYRLIHCNTSTIAQACPKVNFDPIPIYYCAPAGYAILKCNNKTFNGTGPCNNVSTVQCTHGIKPVVSTQLLLNGSLAEEEIVIRSEDLTNNAKTIIVHLKESVEIECTRPGNNTRKSVRIGPGQTFYATGEVIGDIRQAHCNINREKWNKTLQKVGEKLYRHFPNNNTIVFAPSSGGDLEITTHSFNCRGEFFYCNTSALFNSTWQCNNTLCSWVSNNTDKFSNIILYCRIKQIINMWQGVGRAMYASPIAGNITCRSNITGILLTWDGGNSDNNTFRPGGGNMKDNWRSELYKYKVVEIKPLGIAPTGAKRRVVERGKRAVTLGAVFLGFLGAAGSTMGAASITLTVQARQLLSGIVQQQSNLLRAIEAQQHLLQLTVWGIKQLQARVLAIERYLQDQQLLGIWGCSGKLICTTNVRWNSSWSNKTYNEIWNNMTWMQWDKEINNYTGLIYSLLEESQKQQENNEKDLLALDSWQNLWNWFDISKWLWYIKIFIMIVGGLIGLRIIFAVLSIVNRVRQGYSPLSFQTLIPNPRGPDRLERIEEEGGEQDKGRSIRLVNGFLALIWDDLRNLCLFSYHRLRDFILIAARAAELLGHSSLRGLQRGWEILKYLGSLVQYWGLELKKSAISLLDTIAIAVAEGTDRIIEAAQRIGRAIYNIPRRIRQGFEAALQ | 1:250 | 1:10 | 1:5 | 1:5 | 1:5 |
| Con6 gp120/B | AENLWVTVYYGVPVWKEANTTLFCASDAKAYDTEVHNVWATHACVPTDPNPQEIVLENVTENFNMWKNNMVEQMHEDIISLWDQSLKPCVKLTPLCVTLNCTNVRNVSSNGTETDNEEIKNCSFNITTELRDKKQKVYALFYRLDVVPIDDKNSSEISGKNSSEYYRLINCNTSAITQACPKVSFEPIPIHYCAPAGFAILKCNDKKFNGTGPCKNVSTVQCTHGIKPVVSTQLLLNGSLAEEEIIIRSENITNNAKTIIVQLNESVEINCTRPNNNTRKSIHIGPGQAFYATGEIIGDIRQAHCNISRTKWNKTLQQVAKKLREHFNNKTIIFKPSSGGDLEITTHSFNCGGEFFYCNTSGLFNSTWMFNGTYMFNGTKDNSETITLPCRIKQIINMWQGVGQAMYAPPIEGKITCKSNITGLLLTRDGGNNSNKNKTETFRPGGGDMRDNWRSELYKYKVVKIEPLGVAPTKAKRRVVEREKR | 1:250 | 1:10 | 1:5 | 1:5 | 1:5 |
| ConS gp140 | AENLWVTVYYGVPVWKEANTTLFCASDAKAYDTEVHNVWATHACVPTDPNPQEIVLENVTENFNMWKNNMVEQMHEDIISLWDQSLKPCVKLTPLCVTLNCTNVNVTNTTNNTEEKGEIKNCSFNITTEIRDKKQKVYALFYRLDVVPIDDNNNNSSNYRLINCNTSAITQACPKVSFEPIPIHYCAPAGFAILKCNDKKFNGTGPCKNVSTVQCTHGIKPVVSTQLLLNGSLAEEEIIIRSENITNNAKTIIVQLNESVEINCTRPNNNTRKSIRIGPGQAFYATGDIIGDIRQAHCNISGTKWNKTLQQVAKKLREHFNNKTIIFKPSSGGDLEITTHSFNCRGEFFYCNTSGLFNSTWIGNGTKNNNNTNDTITLPCRIKQIINMWQGVGQAMYAPPIEGKITCKSNITGLLLTRDGGNNNTNETEIFRPGGGDMRDNWRSELYKYKVVKIEPLGVAPTKAKLTVQARQLLSGIVQQQSNLLRAIEAQQHLLQLTVWGIKQLQARVLAVERYLKDQQLEIWDNMTWMEWEREINNYTDIIYSLIEESQNQQEKNEQELLALDKWASLWNWFDITNWLW | 1:2,500 | 1:10 | 1:20 | 1:5 | 1:5 |
| 1086C gp140 | SWVTVYYGVPVWKEAKTTLFCASDAKAYEKEVHNVWATHACVPTDPNPQEMVLANVTENFNMWKNDMVEQMHEDIISLWDESLKPCVKLTPLCVTLNCTNVKGNESDTSEVMKNCSFKATTELKDKKHKVHALFYKLDVVPLNGNSSSSGEYRLINCNTSAITQACPKVSFDPIPLHYCAPAGFAILKCNNKTFNGTGPCRNVSTVQCTHGIKPVVSTQLLLNGSLAEEEIIIRSENLTNNAKTIIVHLNESVNIVCTRPNNNTRKSIRIGPGQTFYATGDIIGNIRQAHCNINESKWNNTLQKVGEELAKHFPSKTIKFEPSSGGDLEITTHSFNCRGEFFYCNTSDLFNGTYRNGTYNHTGRSSNGTITLQCKIKQIINMWQEVGRAIYAPPIEGEITCNSNITGLLLLRDGGQSNETNDTETFRPGGGDMRDNWRSELYKYKVVEIKPLGVAPTEAKERVVEREKEAVGIGAVFLGFLGAAGSTMGAASMTLTVQARQLLSGIVQQQSNLLRAIEAQQHMLQLTVWGIKQLQARVLAIERYLKDQQLLGMWGCSGKLICTTAVPWNSSWSNKSQNEIWGNMTWMQWDREINNYTNTIYRLLEDSQNQQEKNEKDLLALDSWKNLWNWFDISKWLWYIK | 1:2,500 | 1:10 | 1:20 | 1:5 | 1:5 |
| Rec MN gp41 | TVQARLLLSGIVQQQNNLLRAIEAQQNMLQLTVWGIKQLQARVQAVERYLKDQQLLGFWGCSGKLICTTTVPWNASWSNKSLDDIWNNMTWMQWEREIDNYTSLIYSLLEKSQTQQEKNEQELLGLDKWESLWNWFDITNWLENRVRQGYSPLSLQTRPPVPRGPDRPEGIEEEGGERDRDTSGRLVHGFLAIIWVD | 1:12,500 | 1:10 | 1:20 | 1:5 | 1:5 |
| A1.con env03 gp140 | AENLWVTVYYGVPVWKDAETTLFCASDAKAYETEMHNVWATHACVPTDPNPQEIHLENVTEEFNMWKNNMVEQMHTDIISLWDQSLKPCVKLTPLCVTLNCSNVNVTNNTTNTHEEEIKNCSFNMTTELRDKKQKVYSLFYRLDVVQINENNSNSSYRLINCNTSAITQACPKVSFEPIPIHYCAPAGFAILKCKDKEFNGTGPCKNVSTVQCTHGIKPVVSTQLLLNGSLAEEEVIIRSENITNNAKTIIVQLTKPVKINCTRPNNNTRKSIRIGPGQAFYATGDIIGDIRQAHCNVSRSEWNKTLQKVAKQLRKYFKNKTIIFTNSSGGDLEITTHSFNCGGEFFYCNTSGLFNSTWNNGTMKNTITLPCRIKQIINMWQRAGQAMYAPPIQGVIRCESNITGLLLTRDGGNNNTNETFRPGGGDMRDNWRSELYKYKVVKIEPLGVAPTRAKTLTVQARQLLSGIVQQQSNLLRAIEAQQHLLKLTVWGIKQLQARVLAVERYLKDQQLLGIWGCSGKLICTTNVPWNSSWSNKSQNEIWDNMTWLQWDKEISNYTHIIYNLIEESQNQQEKNEQDLLALDKWANLWNWFDISNWLW | 1:2,500 | 1:10 | 1:5 | 1:5 | 1:5 |
| B.con env03 gp140 | AEKLWVTVYYGVPVWKEATTTLFCASDAKAYDTEVHNVWATHACVPTDPNPQEVVLENVTENFNMWKNNMVEQMHEDIISLWDQSLKPCVKLTPLCVTLNCTDLMNATNTNTTIIYRWRGEIKNCSFNITTSIRDKVQKEYALFYKLDVVPIDNDNTSYRLISCNTSVITQACPKVSFEPIPIHYCAPAGFAILKCNDKKFNGTGPCTNVSTVQCTHGIRPVVSTQLLLNGSLAEEEVVIRSENFTDNAKTIIVQLNESVEINCTRPNNNTRKSIHIGPGRAFYTTGEIIGDIRQAHCNISRAKWNNTLKQIVKKLREQFGNKTIVFNQSSGGDPEIVMHSFNCGGEFFYCNTTQLFNSTWNGTWNNTEGNITLPCRIKQIINMWQEVGKAMYAPPIRGQIRCSSNITGLLLTRDGGNNETEIFRPGGGDMRDNWRSELYKYKVVKIEPLGVAPTKAKTLTVQARQLLSGIVQQQNNLLRAIEAQQHLLQLTVWGIKQLQARVLAVERYLKDQQLLGIWGCSGKLICTTAVPWNASWSNKSLDEIWDNMTWMEWEREIDNYTSLIYTLIEESQNQQEKNEQELLELDKWASLWNWFDITNWLW | 1:2,500 | 1:10 | 1:5 | 1:5 | 1:5 |
| gp70 V1V2 1086.C | CTNVKGNESDTSEVMKNCSFKATTELKDKKHKVHALFYKLDVVPLNGNSSSSGEYRLINC | 1:100 | 1:10 | 1:5 | 1:5 | 1:5 |
| Bio-V3.C | KKKNNTRKSIRIGPGQTFYATGDIIGDIRQAHC | 1:2,500 | 1:10 | 1:5 | 1:5 | 1:5 |
| gp70 ConC V3 tags | CTRPNNNTRKSIRIGPGQTFYATGDIIGDIRQAHC | 1:250 | 1:10 | 1:5 | 1:5 | 1:5 |
| gp70 B.CaseA V1V2 | CIDLRNATNATSNSNTTNTTSSSGGLMMEQGEIKNCSFNITTSIRDKVQKEYALFYKLDIVPIDNPKNSTNYRLISC | 1:100 | 1:10 | 1:5 | 1:5 | 1:5 |
