# Supplementary material for: Maternal Humoral Immune Responses Do Not Predict Postnatal HIV-1 Transmission Risk in Antiretroviral-Treated Mothers from the IMPAACT PROMISE Study
Source: mSphere. 2019 Oct 23;4(5):e00716-19. doi: 10.1128/mSphere.00716-19 (PMC7407004; doi:10.1128/mSphere.00716-19)
Supplement: TABLE S3 [file mSphere.00716-19-st003.docx]

|  |  | **Transmission Status** | |
| --- | --- | --- | --- |
| **Characteristic** |  | **Transmitter (N=19)** | **Non-Transmitter (N=57)** |
| Breast milk total IgA against HIV-1 B.con env03 gp140 (AUC) | Min-Max | 0.21-2.56 | 0.17-5.45 |
|  | Mean (s.d.) | 0.85 (0.66) | 0.84 (1.16) |
|  | Median (Q1-Q3) | 0.73 (0.37-1.06) | 0.40 (0.27-0.71) |
| Breast milk sIgA against HIV-1 B.con env03 gp140 (AUC)^a^ | Min-Max | 0.25-2.62 | 0.20-4.38 |
|  | Mean (s.d.) | 0.85 (0.58) | 0.71 (0.86) |
|  | Median (Q1-Q3) | 0.65 (0.50-1.18) | 0.42 (0.29-0.65) |
| Breast milk ADCC antibody titer^a^ | Below Lower Limit of Detection | 5 (26%) | 23 (40%) |
|  | Detectable | 14 (74%) | 34 (60%) |
| Plasma total IgA against HIV-1 B.con env03 gp140 (AUC) | Min-Max | 0.22-5.43 | 0.20-7.66 |
|  | Mean (s.d.) | 1.40 (1.34) | 1.12 (1.58) |
|  | Median (Q1-Q3) | 1.02 (0.44-1.83) | 0.48 (0.32-1.03) |
| Plasma ADCC antibody titer^a^ | Below Lower Limit of Detection | 6 (32%) | 18 (32%) |
|  | Detectable | 13 (68%) | 39 (68%) |
| Breast milk ADCC potency (maximum % specific killing) | Min-Max | 14.00-39.97 | 0.00-49.80 |
|  | Mean (s.d.) | 26.38 (8.01) | 24.66 (13.26) |
|  | Median (Q1-Q3) | 27.66 (17.78-32.04) | 25.86 (14.03-34.10) |
| Plasma ADCC potency (maximum % specific killing) | Min-Max | 15.90-42.84 | 6.42-39.12 |
|  | Mean (s.d.) | 26.29 (8.69) | 24.41 (8.67) |
|  | Median (Q1-Q3) | 26.06 (19.19-30.72) | 25.00 (17.99-30.42) |
